# Green Corrosion Inhibitors from Natural Sources and Biomass Wastes

**DOI:** 10.3390/molecules24010048

**Published:** 2018-12-23

**Authors:** Stefania Marzorati, Luisella Verotta, Stefano P. Trasatti

**Affiliations:** Department of Environmental Science and Policy, Università degli Studi di Milano, via Celoria 2, 20133 Milano, Italy; luisella.verotta@unimi.it (L.V.); stefano.trasatti@unimi.it (S.P.T.)

**Keywords:** green chemistry, metal protection, corrosion inhibitors, natural products, biomass waste

## Abstract

Over the past decade, green chemistry has been emphasizing the importance of protecting the environment and human health in an economically beneficial manner aiming at avoiding toxins and reducing wastes. The field of metallic materials degradation, generally faced by using toxic compounds, found a fertile research field in green chemistry. In fact, the use of inhibitors is a well-known strategy when metal corrosion needs to be prevented, controlled, or retarded. Green inhibitors are biodegradable, ecologically acceptable and renewable. Their valorization expands possible applications in industrial fields other than ‘waste to energy’ in the perspective of circular economy. Although lot of experimental work has been done and many research papers have been published, the topic of green inhibitors is still an open issue. The great interest in the field expanded the research, resulting in high numbers of tested molecules. However, the most frequently adopted approaches are conventional and, hence, not suitable to fully characterize the potential efficacy of inhibitors. All the mentioned aspects are the object of the present review and are meant as a constructive criticism to highlight the weak points of the green inhibitors field as to re-evaluate the literature and address the future research in the field that still lacks rationalization.

## 1. Introduction

In the last decade, green chemistry has been attracting great interest in many contexts by designing chemicals, chemical technologies, and commercial products with the aim to avoid toxins and reduce wastes. Already in the 1980s, there was a growing concern regarding the generation of waste and the use of toxic and hazardous chemicals [1]. The stimulus for a turning point in the environmental awareness was provided by the announcement of the Pollution and Prevention Act in 1990 by the USA [2]. Environmental issues and their solutions became a priority all over the world. Waste prevention from being formed at the origins, together with source reduction programs and many other related principles have been announced by the “12 Principles of Green Chemistry” formulated by Anastas a few years later [3]. The principles were meant as guidelines and design criteria by molecular scientists.

Since then, green chemistry has grown into a significant internationally engaged focus area within chemistry. Many countries inside and outside Europe such as Italy, the United Kingdom and Japan, launched major initiatives in green chemistry. Several research, education, and outreach programs were adopted as central themes [4]. Over the years, green chemistry was able to demonstrate how fundamental scientific methodologies can protect human health and the environment in an economically beneficial manner incorporating areas such as polymers [5], solvents [6], catalysis [7], analytical method development [8], synthetic methodology development [9] and, in general, the design of safer chemicals with an increased awareness for environmental impact. In this context, biomass and renewable raw materials are at the basis of the alignment of many industrial applications to the principles of green chemistry and sustainability. Since Nature provides an incredibly wide range of matrices with variable chemical compositions, the interest in renewable raw materials has been growing. Various forms of biomass are also essential for human and animal nutrition and this means that their use for other purposes must be balanced. Ideally, the biomass remaining after removal of the nutritious components, the bio-waste, can serve as a feedstock for a number of applications that might be related to green chemistry [10,11,12,13].

One of the fields in which green chemistry, often applied to biomass-derived product, is providing innovation, in terms of reducing the environmental impact and the wastes, is related to the metallic surface protection. Metallic material degradation has been generally faced by the use of environmentally unfriendly organic compounds. Acidic and aggressive solutions are widely used in process industries for the removal of deposited scales from metallic surfaces during the production, exercise and storage stages [14]. The use of inhibitors is a well-known strategy when metal corrosion needs to be controlled, prevented or retarded [15]. 

Although most of organic molecules tend to adsorb on metal surfaces, usually, when aiming at the corrosion inhibition effect, the most desirable features are specific and related to: the metal electrostatic attraction on the charged inhibitor molecules; the interaction of double/triple bonds or lone pair electrons of (N, S, P or O) with the vacant d-orbital of the metal; or a combination of some of the previous mechanisms [16]. As examples, a number of heterocyclic synthetic compounds have been reported as corrosion inhibitors and the screening of synthetic heterocyclic compounds is still being continued [17]. Although many synthetic compounds showed good anticorrosive activity, a global concern arose because of their toxicity to both humans and environment either during the synthesis of the compound or its applications [18,19]. The concern is not only related to the environmental poisoning, needing effective procedures of inhibitor removal before flowing the used solution out of the industrial plant, but also to the contact of the protected metal for example with food, beverage, medicals, during its lifecycle. These inhibitors may cause temporary or permanent damage to organ system, such as kidney or liver, or to disturb a biochemical process or an enzyme system at some site in the body [20]. Vermeirssen and co-workers evaluated the release of toxic substances, such as Bisphenol A, used as component in resins to protect the integrity and functioning of steel [18]. They tested samples for: the agonistic and antagonistic effects on various mammalian nuclear receptors; inhibition of photosynthesis and growth in algae; inhibition of bacterial bioluminescence; and inhibition of water flea reproduction. Results showed that anti-corrosion coatings based on those resins could be a source of toxicity to the aquatic environment.

**Figure 1:**
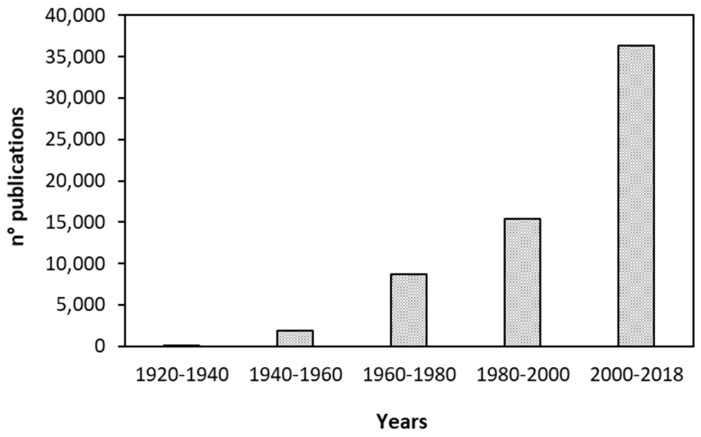
Number of publications per 20-year-range from 1920 to 2018.

Corrosion of metals and alloys is an important and well-studied industrial problem which has hence found a fertile research field in green chemistry. Green inhibitors are attracting great interest in the corrosion field thanks to their safety, biodegradability, ecologically acceptability and renewability [21]. These include for examples amino acids [22], alkaloids [23], polyphenols [24] and often extracts of plants [25] largely distributed and of low economic value, including byproducts of agro-industrial processes and agricultural-wastes [12]. Their valorization expands possible applications in industrial fields other than “waste to energy” in the perspective of circular economy. The literature around the topic of green corrosion inhibitors for these metal surfaces is particularly active, as demonstrated in Figure 1. The number of published papers (patents included) over the topic “green corrosion inhibitors”, as obtained through a SciFinder^®^ literature review, is represented in the figure. The increase of publication shows an exponential trend. 

However, many weak points arise from a careful re-evaluation of them in a broader context. Although a lot of experimental work has been done and many research papers have been published in the field, there is still a lack in the scientific approach, resulting often phenomenological and rarely ever theoretical and mechanistic. Testing is generally conventional and performed by electrochemical experiments, aiming at directly identifying the efficiency in the final application as corrosion protective agents rather than setting the focus on the specific adsorption models and involved mechanisms [26,27]. Another non-negligible weakness is related to the limited characterization of the active compound in the green extract, which often comprises a mixture of unidentified molecules. This worsens a field in which the reproducibility of results strongly depends on the harvesting location, weather conditions, season, harvesting technique and many other parameters. Sampling procedures might be crucial and lead to conflicting results. As an example, Fouda et al. prepared an aqueous extract of propolis to evaluate the corrosion inhibition on carbon steel [28]. The results obtained showed that propolis could serve as an effective inhibitor for the corrosion of carbon steel in aqueous media. Inhibition was found to increase with increasing concentration of the extract providing an inhibition efficiency of up to 92%. A few years later, another work published by Dolabella and co-workers, used a commercial ethanolic extract of the same material as corrosion inhibitor for the same metal, carbon steel [29]. Although the tested matrix was the same (propolis), obtained by different extraction techniques (aqueous or ethanolic extractions), and even if the two works claimed positive results in terms of effectiveness of inhibition, from the quantitative point of view, experiments provided completely different results. In addition, the inhibition effect might be related to one specific molecule or to a synergistic effect of the entire extract. This aspect is not usually taken into account in much of the literature.

This review is meant as a collecting overview of significant works in the literature focusing on natural products and biomass wastes employed as green corrosion inhibitors. A special emphasis is given to highlight the weak points and deficiencies of the green inhibitors field as a constructive criticism to re-evaluate the literature and address future research in the field that sometimes lacks in rationalization. All the extracts employed as green corrosion inhibitors that will be mentioned in the paper are listed in Table 1. The table summarizes their relative natural source, the extraction methodology, the protected metal and the corrosive environment, the inhibition efficiencies and other useful information.

## 2. Economic and Industrial Opportunities

As first aspect to be revised, an analysis of the costs behind corrosion prevention is needed in order to understand if the inhibition is an economically favorable action. Since the 1950’s several countries considered the economic consequences of corrosion. The phenomenon of corrosion affects the technological process and many aspects of human life, causing directly or indirectly damages to the economy. Several industries have come to realize that lack of corrosion management can be very costly and that, through proper corrosion management, significant cost savings can be achieved. Already in 1978, a report from the National Bureau of Standards (USA) listed the cost items involved as direct damages of corrosion [30]. These included: the reconstruction of civil or industrial buildings and the substitution of machines and plants; the product losses, due to corrosion occurring during their production; the maintenance; the production redundancy, to compensate the production stops due to corrosion processes; the machines redundancy, to minimize the production stops; the use of inhibitors and other protection actions; research in the field; insurances; workers training; salary of people in charge of check and maintenance. A compromise between corrosion occurrence and anti-corrosion action should be figured out in a way that the latter would be able to affect the involved costs in a reasonable way. 

Talking with numbers, NACE^®^ International (National Association of Corrosion Engineers), a worldwide corrosion authority, reported that the global cost of corrosion was estimated to be US$2.5 trillion, which is equivalent to 3.4% of the global gross domestic product (2013). By using available corrosion control practices, it is estimated that savings of between 15 and 35% of the cost of corrosion could be realized; i.e., between US$375 and $875 billion annually on a global basis [31]. This provides wide breath in the field also in the corrosion inhibition field.

Among the great number of patents related to corrosion prevention actions and corrosion inhibitors uses, however, just few of them directly deal with green corrosion inhibitors. From an analysis of the patents literature in this specific field, one of the found application is for the use of *Phoenix clactylifera* seeds as corrosion inhibitor for steel and in general for metals [32]. The work highlighted also the economical aspect focusing on the bio-waste, pointing out that the fruit of this plant is a one seeded berry. The pericarp of the fruit is edible and highly nutritious. The seed, thrown as waste, was the one examined for its corrosion inhibition properties. The inhibition efficiency was indeed around 97% at 50 °C. Another patent application found in the literature, in the field of the prevention of the microbiologically influenced corrosion, responsible for 40% of the internal corrosion problems in oil transportation pipelines, storage tanks, water-cooling system and fire protection systems, tested plant-derived antimicrobial agents as corrosion inhibitors. The cited composition included extract of different part of the plants such as lantana, tobacco, neem, mahendi, black pepper, red chili, vegetable and/or fruit plant material and/or mixtures. Plant material included the stem, leaves and fruit of the plant and any part of the plant [33]. 

In a Chinese patent, corrosion inhibitor of sweet potato stems and lettuce flower stalks were employed as to provide biodegradable, environmentally-friendly products with a reduction of pollution, excluding the conventional synthesis inconveniences [34]. An invention by Gomes et al. was related to the use of fruit skin extracts (such as mango, cashew, passion-fruit and orange) as corrosion inhibitors for steel in an acid medium [35]. The inventors pointed out, in this case, the importance of working on wastes, since they selected skin and seeds, byproducts of fruit juices.

Another patent was instead addressed to methods, systems and apparatuses for predictively selecting plant extracts for their inclusion in formulations to impart anti-corrosion properties to coatings for aluminum, aluminum alloys, copper and copper alloys [36]. A last example is provided by Versalis, the chemical department of the Italian petrochemical giant Eni, and Elevance Renewable Sciences, a US-based producer of specialty products based on natural oils. Together, in 2014 they announced their project to develop a new technology to produce chemical products based on vegetable oils. Their patent, deposited the last year, deals with corrosion inhibitors from vegetable oils [37] in a more general biorefinery context, providing green innovation and boosting the transformation of petrochemical sites into more sustainable facilities, while creating synergies between renewable resources and the petrochemical industry. 

A weak aspect of the field of corrosion inhibitors from natural products is the scarce application in real-scale plants for damages prevention. This aspect could be related to the generally adopted mistake of deriving green products as inhibitors from fruits, vegetables or matrices that are valuable because they are edible, scarce or hard to be harvested. This is pointed out in Table 1, where it comes out that researchers did not show any preference of working on extracts from bio-wastes. The lab-scale research literature is allowed to work easily on every kind of natural sample from which an extract can be in principle derived, no matter if the source is valuable. Chances that the extract could work as corrosion inhibitor are quite high, considering that the main properties connected to the corrosion inhibition are the presence of heteroatoms and/or unsaturated bonds. These features are in fact quite common in natural extracts. Even if this kind of “pure” research is not in principle asked to consider the economical/social impact of its work, it is however worth pointing out the importance of the final application. 

If the previously mentioned numbers of economical savings are true, this means that researchers should rather focus on specific and selected matrices and sources. These include bio-wastes in general and not matrices characterized by valuable nutritional aspects or natural scarcity. A full research procedure, starting from reasonable sources from waste, including a characterization of the extract and an identification of the main compound/s responsible of the inhibition and theoretical and mechanistic considerations, could provide an important added value to this field.

## 3. Corrosion

The wide range of constructional materials available to innovative technologies has broadened the series among which they are selected in order to choose the most appropriate to each application on the basis of its mechanical or physical properties. Metallic materials play a key role in the development of a country and its sustained growth in the context of the global economy. However, there is no application where the effect of the interaction of a metal or alloy with its environment can be completely ignored. The corrosion issues are nowadays debated in many contexts [38], from the suitability over years and ageing of drinking water pipes, to the oil and gas distribution, to the monitoring or post-disaster considerations related to infrastructures failures [39]. 

On the basis of IUPAC definition, corrosion is an irreversible interfacial reaction of a material (metal, ceramic, polymer) with its environment which results in consumption of the material or in dissolution of a component of the environment in the material. Often, but not necessarily, corrosion results in effects detrimental to the usage of the material. Exclusively physical or mechanical processes such as melting or evaporation, abrasion or mechanical fracture are not included in the term corrosion [40]. 

Corrosion reactions are often intrinsically electrochemical. The most common reactions, sustaining a corrosion process, involve the hydrogen evolution reaction (common in acids media) and oxygen reduction reaction (in neutral or alkaline environments). The most important factors involved in corrosion are connected to: the metal itself, in terms of its composition, atomic structure, micro and macroscopic defects, stress response (tensile, compressive, cyclic); the environment and its chemical nature, quality and quantity of reactive species, pressure, temperature, etc.; the metal-environment interface, kinetics of metal oxidation and dissolution and kinetics of the reduction of species in solution [41]. 

In order to prevent or limit corrosion phenomena, the first option is to select the appropriate metal or alloy (e.g. changing the surface composition by surface oxide formation) on the basis of its final application and the other necessities. Secondly, a control of the environmental parameters (temperature, pH, etc.) and an eventual addition of specific inhibitors concentration should be optimized. 

Among the large number of different metal and metal alloys that need more than others to be protected from corrosion, due to their specific applications, steels, aluminum and its alloys, zinc and copper alloys are at the core of the research and applied corrosion protection issue. 

Due to their excellent and wide range of mechanical properties, steels are widely used in chemical and oil & gas industries, thus requiring protection from corrosion for engineering, automobile, boiler plates, pipes, reaction vessels, storage tanks and in bridges and building works; since steel undergoes severe corrosion attack in aggressive environment, it needs to be protected [42,43,44,45,46,47]. Also, corrosion of the steel reinforcement is one of the main reasons causing the premature deterioration of reinforced concrete, and leading to a significant economic loss or serious accidents [48]. Stimulated by the warmth of the topic, starting from preliminary results, Fiori-Bimbi and co-workers employed pectin alone as an efficient eco-friendly corrosion inhibitor for mild steel in hydrochloric acid solutions [16]. Pectin was extracted from citrus peel as raw material. Its extraction procedure comprised first the size reduction of fresh lemon peel to below 1 cm thick. Acid hydrolysis extraction step was performed using a boiling hydrochloric solution of pH 2 for 90 min. Pectin was precipitated from the filter liquor with ethanol, then filtered, repeatedly washed and dried. Their work gained a deep insight into the mechanistic aspects of the corrosion inhibition process by pectin, discussing fundamental aspects that are often ignored in the literature on this subject matter. Considering the commercial molecule “pectin” as corrosion inhibitor of steel, other works, on the other hand, highlight its effectiveness in combination with other compounds, such as cerium oxide, claiming high performances, however, not supported by impressive results in corrosion inhibition neither by deep data discussion [26,27]. Some weak points of the cited papers are related also to the unknown reproducibility of the electrochemical tests. The reported data are pretty similar one to the other, and the claimed differences should be proofed by some statistical analyses which are unfortunately missing. This lack is quite common in the field [49].

Aluminium is considered another metal at the center of the focus of corrosion protection [22,50,51,52,53,54]. It serves a multitude of purposes, starting from foils for wrapping, through every engineering industry to high technological applications in aeronautics, space exploration, power sources and electronics [51]. Most aluminium alloys have good corrosion resistance towards natural atmospheres and other environments, because aluminium alloys surfaces are covered with a natural oxide film of thickness about 5 nm. However, the oxide film is generally dissolved in strong acids and bases. In the presence of aggressive ions, like chloride, the protective layer can be locally destroyed and corrosive attack takes place. The most commonly used alloys in aircraft manufacturing are Al 2024 and 7075. Pitting corrosion in the presence of chloride ions is often encountered cause of failure of these high-strength aluminum alloy [22]. Yet, if correctly protected, applications of aluminium alloy may be more reliable and have long service life [52]. Aluminium protection by eco-friendly composites is another hot topic in this research field. A multitude of plant extracts have been studied as environmental-friendly corrosion inhibitors [55,56,57]. One more time, pectin from citrus peel was employed as green inhibitor also for aluminium corrosion protection [58]; the characterization of the process was conducted as routine, by weight loss measurements. An attempt to build an adsorption model was made. A Langmuir-type adsorption model was used to fit experimental data. It is of particular concern that most of the adsorption models used in the literature of the field, independently from the metallic substrate or the inhibitor chemistry, are fit using the Langmuir one [45,53,59]. This needs to be revised, since the Langmuir model assumptions are far from the reality, as it will be discussed later. No other fitting attempts were presented. 

Corrosion protection of copper and zinc alloys acquires a sectorial importance when considering the historical manufactures, ancient and historical artworks and their conservation over years [60]. The degradation of most metals, with the exception of gold is a universal reaction and it constitutes a constant and on-going issue in fields such as heritage conservation or other industrial applications related to the metals electrical and thermal conductivities [61,62]. Metals from cultural heritage may suffer from degradation phenomena during the archaeological burial, from indoor atmospheric corrosion during exhibition or storage in uncontrolled environmental conditions and from outdoor atmospheric degradation in the case of buildings or statues [63]. Laboratories tests using aerated weakly acidic solution containing Na_2_SO_4_ and NaHCO_3_ at pH = 5 are for example carried out in order to simulate acid rain in an urban environment for the protection of bronze artifacts [59]. Another example of protection of bronze statues, by an eco-friendly inhibitor from *Salvia Hispanica*, was performed by Larios-Galvez et al. [60]; even if the novelty of the paper is the application field that is quite sectorial and not so popular as others (i.e., the steel corrosion protection by green inhibitors), the main defect is the lack of mechanistic understanding of the involved protection mechanism, starting from a complete characterization of the single compounds in the extract. 

## 4. Corrosion Inhibitors

On what concerns the corrosion inhibitors, that is the object of the present review, they are substances added in low concentration to the process fluid, able to slow down the corrosion reactions. Sometimes their inhibition mechanism is not straight to interpret and study. The inhibition effect can be anodic, cathodic or mixed [64]. It is also worth specifying that some inhibitors are strictly specific to the metal or alloy and environment, and might not show any inhibition phenomenon with others. 

Anodic inhibitors usually act by forming a protective oxide film on the surface of the metal, cathodic inhibitors act by either slowing the cathodic reaction itself or selectively precipitating on cathodic areas to limit the diffusion of reducing species to the surface. 

Most of the green corrosion inhibitors belong to the mixed class [16,65,66,67]. Mixed-type inhibitors are able to accomplish a cathodic and anodic action at the same time by decreasing both their electrochemical rates. The mechanism of inhibition is often related to the adsorption of molecules with double/triple bonds or containing V and VI group elements such as N, P, S, O, which contain free electron couples. The determination of the “class” is quite easy to be carried out. Other insights are instead necessary for the determination of the mechanisms, and this is the main lack in the literature. Satapathy et al. extracted the fresh leaves of *Justicia gendarussa* plant with methanol [68]. By FT-IR spectroscopy they found out the presence of mixtures of compounds, such as sitosterol, friedelin, lupeol etc. and naturally occurring phenolic dimers, O-substituted aromatic amines and flavonoids. Admitting the difficulties of separating them, as commonly occurs in most of the literature works, the methanol extract was used as it was for corrosion inhibition studies. Electrochemical studies indicate that the plant extract acted as mixed-type inhibitor with predominant cathodic effectiveness. However, apart from these data, no clear indication about the mechanism was provided, other than an adsorption fitting performed with a Langmuir model isotherm, which is often intrinsically not correct, as it will be discussed later in the review. The plot relative to the fitting would be in fact clearly better fit with a non-linear model. Moreover, since the extract comprises a mixture of molecules of different nature, it would also be complicated to made any trustable hypothesis of a mechanism of the basis of such complexity. El-Haddad et al. published a paper evaluating the corrosion inhibition effect of chitosan, purchased by Sigma Aldrich, onto copper surfaces, hence excluding any parallel effect due to other compounds in the used solution [69]. Their results were more trustable in terms of electrochemical features and they classify the molecule as a mixed-type inhibitor acting preferably on the cathodic sites. Still, they used a Langmuir isotherm model to fit data, but they added a deeper computational study on the HOMO-LUMO orbitals and their hypothesis on the inhibition mechanism is more reliable due to the exact knowledge of the involved molecule, the only one that can play a role in the final inhibition effect. 

Few works found out cathodic-type inhibitors. Banerjee and co-workers tested a polyacrylamide grafted with Okra mucilage, a natural grade polysaccharide, as corrosion inhibitor for mild steel in 0.5 M H_2_SO_4_. They assigned the inhibition mechanism to the cathodic one, even if they admit that this could not be the only way to interpret their experimental data [70]. Gerengi et al. reported a “slightly” cathodic inhibition efficiency of a *Schinopsis lorentzii* extract. However, a statistical analysis is not reported and data need to be trusted without any error bar, even if the curves are very similar to each other [71]. The classification of a specific inhibitor is in fact sometimes not straight. In a work by Khaled, he found some differences depending on the environment: he reported that a guanidine derivative behaves as cathodic in 1 M HCl and as mixed-type in 0.5 M H_2_SO_4_ [72]. An interesting paper was published by Amin et al. [67], evaluating the performance of three selected amino acids, namely alanine, cysteine and S-methyl cysteine as safe corrosion inhibitors for iron in aerated stagnant 1.0 M HCl solutions. Their work was quite well organized in terms or evaluation of the possible mechanisms, also on the basis of computational studies evaluating the HOMO-LUMO orbitals and many hypotheses about modes of adsorption (physical, chemical and adsorption via H-bonding) of the three amino acids on the electrode surface were formulated. In a work studying the inhibition effect of commercial pectin X60 pipeline steel in acid medium [26], authors pointed to pectin functioning as the mixed type inhibitor with predominant control of the cathodic reaction. This sometimes happens with mixed-type inhibitors, with a more pronounced effect onto one of the two redox reactions occurring.

A last class is relative to anodic inhibitors, able to increase the anodic process overpotential, favoring a film formation on the metallic surface. They form a protective layer of oxide film on the surface of metal, causing an anodic shift of the corrosion potential resistance. On what concerns the eco-friendly anodic inhibitors, already in 1988 Sekin et al. investigated the inhibition mechanism of Vitamin C and folic acid over steel in NaCl solution [73]. Their results went in the direction of an anodic-type inhibition, which were later, in 2004, contested by Ferreia et al. [66]. They instead claimed a mixed-type inhibition effect. However, all these considerations were based on small potential and current value shifts and without any support on deeper mechanistic considerations. 

The basis of the previous classification is the final inhibition effect, depending if it affects the anodic, cathodic or both the reaction rates. However, beyond and before the final observed phenomenon, a corrosion inhibition mechanism should be investigated and studied. Over the years, the approach of many research groups had been focused on the final phenomenological effect rather than studying the relations between the molecular structure and the corrosion inhibition.

Already in 1997, when most of the corrosion inhibitors were still of organic origin, in a NACE International^®^ report, Sastri pointed out how the selection of these inhibitors had been based on an empirical approach to date. Even if many attempts have been made to correlate inhibition efficiency with properties of the organic molecules, for proper selection of inhibitors, mechanistic information on corrosion and inhibition processes is still a lack in many literature works [74].

Generally, the corrosion inhibition mechanism is adsorption of the inhibitor on the metal surface. The process of adsorption is influenced by different factors like the nature and charge of the metal, the chemical structure of the organic inhibitor (functional groups, aromaticity, possible steric effects, etc.) and the type of aggressive electrolyte [75]. Some decisive factors contributing to the effectiveness of corrosion inhibition are: the size of the organic molecule, the aromaticity and/or conjugated bonding, carbon chain length, bonding strength to metal substrate, the type and number of bonding atoms or groups in the molecule, the ability for a layer to become compact or cross-linked, the ability to form a complex with the atom as a solid within the metal lattice, and adequate solubility in the environment [25]. Most of the effective inhibitors are organic compounds containing N, S, or O atoms in their structures, functional electronegative groups and π electrons in triple or conjugated double bonds. The inhibiting action of these organic compounds can be ascribed to their interactions with the metal surface via an adsorption process. 

Most usually, adsorption of organic compounds on the metal surface takes place due to: the metal electrostatic attraction on the charged inhibitor molecules; the interaction of lone pair electrons of heteroatoms with the vacant d-orbital of the metal; or a combination of some of the previous mechanisms [16]. Consequently, inhibitors adsorption is first dependent on the nature and surface charge of the metal. For all the above-mentioned factors, it is quite clear that the inhibition mechanism should be carefully studied because it provides a quantity of information that is mandatory to take into consideration when working in the field of corrosion inhibition of specific metals in specific environments.

Many research groups focus their research on crude extracts, missing the specificity of action of single compounds, both in the final effect and in the physico-chemical explanation of the involved mechanisms. Oguzie et al., working on the corrosion inhibition effect of acidic extracts of *Piper guineense*, pointed out the importance of working on extracts from biomasses, due to the abundant presence of inexpensive, non-toxic and readily available phytochemical constituents obtained from renewable sources. However, they also highlighted that, even if crude biomass extracts, comprising of number of organic compounds, should overcome the specificity of action of single compounds, mechanistic insights are lacking in many works in the literature. The same authors proposed to overcome some restrictions imposed by the experimental determination of the single contributions of the different constituents, at least mechanistically, by performing theoretical computations in the framework of the density functional theory (DFT) [76]. Identifying piperine, safrole and dihydrocubebin as the main phytochemical constituents present in the *Piper guineense* extract, they built up a model investigating the noncovalent adsorption of molecules on Fe(110), together with additional geometry optimization using the NDDO (neglect of diatomic differential overlap) method with the AM1 Hamiltonian. Other works were able to make some speculations on the adsorption mechanism and hence the inhibition mechanism, lacking however in deeper explanations supported by theoretical and/or experimental data. The corrosion inhibition effect of a Henna extract was investigated by El-Etre et al. Probable interpretation of the observed inhibition action was given without any strong data support, providing hypothesis relatively to complexes formation rather than adsorption mechanism [49]. In another work on Henna extract, Ostovari et al. highlighted the importance of investigating the mechanism beyond the corrosion inhibition effect [77]. They chose conductimetric titration as a reliable procedure to confirm the hypothesis of formation of insoluble complex compounds combined with the metal cations and adsorbed lawsone molecules. In addition, they measured separately the inhibition efficiencies of the constituents of the extract and of the extract itself. Lawsone displayed the highest inhibition efficiency among the other henna constituents such as gallic acid, glucose and tannic acid; it also had a higher inhibition efficiency than the henna extract itself [77].

Streptomycin was also employed as inhibitor and the authors of the work claimed to have obtained an inhibition effect that occurs through adsorption of the drug on metal surface without modifying the mechanism of corrosion process. However, even in this example, the mechanism was not studied but just some hypotheses have been provided [78]. Other previous works investigated the corrosion inhibition effect of other antibacterial drugs such as ampicillin, cloxacillin, flucloxacillin and amoxicillin [79]. The inhibition process was attributed to the formation of insoluble complex with the metal; this hypothesis was not however supported by specific experiments or theoretical calculations but the reasons of employing antibacterial drugs, considering the high costs of this kind of inhibitor, should have been accurately considered and justified.

### Corrosion Inhibitors Testing

Non-destructive and destructive measuring techniques are used as standard methods to determine corrosion rates in the presence and absence of corrosion inhibitors. 

Even if testing of corrosion inhibitors can be performed by some standard methods, a complete characterization should also include some mechanistic considerations regarding the involved corrosion inhibition mechanism. This last approach is not commonly adopted by the literature, that tends to simply follow routine measurements and make some final hypothesis about the experimentally observed phenomena [49,74,78]. 

The weight loss method in corrosion is the simplest and longest-established technique to calculate the corrosion rate. It involves the exposure of a clean weighed piece of the metal or alloy to the corrosive environment for a specified time followed by cleaning to remove corrosion products and weighing the piece to determine the loss of weight. The rate of corrosion (usually in mm per year) is calculated. When an inhibitor is added, an important parameter to be determined is the inhibitor efficiency that takes into account the corrosion rates in the absence and presence of inhibitor [80]. 

It is fundamental to provide error bars in these results because the presence of defects in the analysed metal sample could greatly affect the analysis. The reproducibility of the measurement should be hence checked. Many works in the literature do not provide any standard deviation with their weight loss measurements. As an example, Ostovari et al. [77] and Al-Sehaibani [81] worked separately on Henna extract as corrosion inhibitors for steel in acidic media. Both of their works, when performing weight loss measurements, do not report any reproducibility test and standard deviations are absent. This makes difficult to make a comparison between their results. 

Electrochemical methods are often used routinely for the evaluation of the efficiency of corrosion inhibitors. Electrochemical methods have the advantages of being quite fast in terms of measurement time and they can provide not only information about the corrosion resistance, but also others mechanistic data that might help in the design of corrosion inhibitors and strategies. 

Potentiodynamic polarizations are destructive monitoring methods able to provide a number of information recorded during anodic or cathodic scans in an electrochemical cell which contains both the metal to be investigated and the environment in which the polarization scan is to be performed. Prior to performing the experiment, the sample surface must be prepared such that the initial condition, or starting point, of the measurement is well-defined and does not vary from test to test. In the resulting current vs. potential plot, many regions or, corresponding for example to the open circuit potential, the active region, the passivation region, gas evolution reactions, are recognizable. Once again, a statistical analysis is fundamental to compare results in the literature. The same group, for example, published two papers on the corrosion inhibition of C38 steel in 1 M HCl in 2011 and 2015 [82,83] by some alkaloids extracts. When performing polarizations on C38 steel, the blank measurement without any inhibitor, gave two different results in terms of current vs potential curves. This kind of results should be double-checked in order to avoid these mistakes. Linear Polarization Resistance is another effective method of electrochemical nature useful to measure the corrosion rate [84] by polarizing the material, typically on the order of ±10 mV, relative to its Open Circuit (OC) potential in a three electrodes configuration. As the potential of the material (set as working electrode) is changed, a current will be induced to flow between the working and counter electrodes, and the material’s resistance to polarization can be found by taking the slope of the potential versus current curve. This resistance can then be used to find the corrosion rate of the material [85]. Electrochemical Impedance Spectroscopy (EIS) is another non-destructive electrochemical technique to measure the polarization resistance. EIS measures the electrochemical response to a small AC voltage applied over a range of frequencies. The electrochemical response is used to build an equivalent circuit, on the basis a reaction mechanism, composed of electrical components. An EIS spectrum takes minutes to run compared with the weeks needed for an exposure test. The main difficulties are related to data interpretation because it includes the building of an equivalent circuit modelling the metal solution interface. As an example, EIS was used for the characterization of propolis extracts as corrosion inhibitors for steel. The equivalent circuits used in the literature were dissimilar [28,29]. These differences, if the circuits have been correctly built, would mean that different interface processes, and hence mechanisms, are taking place. 

From both EIS spectra and potentiodynamic curves, information about the interaction (physical or chemical, single or multilayered, etc.) between the inhibitor and metal surface can be achieved by building adsorption isotherms. In general, all the literature in the field tends to employ a Langmuir-type isotherm [65,86]. Its primary assumptions are: (i) adsorbate molecules attach to the active sites of the adsorbent surface, (ii) Langmuir equation assumes that adsorption is monolayer and (iii) all the sites on the solid surface are equal in size and shape and have equal affinity for adsorbate molecules [87]. The last two conditions are hard to fulfill in the corrosion studies, and this is the main weak point about using the Langmuir model. The more complex adsorption models take into consideration factors such as the surface heterogeneity and the presence of areas having different adsorption energy (Temkin isotherm) or interactions between the adsorbed molecules (Frumkin isotherm). It is clearly difficult to select the most appropriate model taking into considerations all the factors, however, many literature attempts to fit data with the simplest Langmuir model failed [88]. In some works, the Langmuir plot are built just with 3 point data [89]. Results from Satapathy et al., where data fitting with Langmuir isotherms are reported, clearly need a revision and some attempts to fit the same with other models [68]. This is not exclusive for Langmuir isotherm. Some work explicitly selected other adsorption models but even in these cases, e.g., Temkin isotherm, results were not coherent with the final fitting [90,91]. Fdil et al., for example, recently used a Langmuir and Temkin model to fit the adsorption of alkaloids extracts from *Retama monosperma* (L.) Boiss. stems. Temkin isotherm was provided as a not discussed figure in the paper but the model clearly is not the appropriate one to be used. If the fitting is not satisfying, other explanation should be provided, instead of forcing the model to fit. An example can be found in a work by Badawy et al. [92], where different amino acids were employed and studied as corrosion inhibitors for Ni-Cu alloys, many different isotherms (including Langmuir, Frumkin and Temkin ) were tested but in most of the cases, no one of them was able to fit data. This was attributed to an interphase inhibitor behavior, presuming a 3D layer between the corroding substrate and the electrolyte. Such layer generally consists of sparingly soluble compounds of corrosion products and/or inhibitor. 

Determination of the thermodynamic/kinetic parameters for the adsorption of inhibitor can provide useful information about the mechanism of corrosion inhibition [93]. The standard free energy of adsorption (ΔG°_ads_) calculation provides information about the strength of adsorption of the molecule on the surface. High values of ΔG°_ads_ indicate that the compound is strongly adsorbed on metal surface in the corrosive media. The standard adsorption heat (ΔH°_ads_) and the standard adsorption entropy (ΔS°_ads_) can be derived from the previous parameter; if negative values are obtained for ΔH°_ads_, the adsorption of inhibitors molecules is an exothermic process with a physical nature (physisorption). Finally, the Arrhenius equation allows to calculate the activation energy (E_a_*). If values of E_a_* are lower than the threshold value required for chemical adsorption, this confirms the physical adsorption of the inhibitors [93]. These calculations are fundamental in the understanding of the involved mechanisms. 

The ΔG°_ads_ value of 40 KJ·mol^−1^ is usually adopted as a threshold value between chemi- and physisorption. Two different groups working on pectin as green corrosion inhibitor for steel in acidic medium, found values of ΔG°_ads_ of −22.83 KJ·mol^−1^ and −23.58 KJ·mol^−1^ at 308 K [16,94]. Values are pretty similar even if pectin was extracted from citrus peel in one paper and cladodes of *Opuntia Ficus Indica* in the other. 

Most of the studies seem to focus on the corrosion inhibition performance of extracts, claiming very positive results; even though mechanistic insights are lacking. Computational studies and quantum chemical calculations, might be however, quite necessary to study the electronic parameters [69,95]. 

Computational evaluations are fundamental in the interpretation of experimental data in order to investigate the mechanisms of corrosion inhibition. After the experiments a computational study, again considering one molecule by one, would be necessary to complete the work. Many papers highlight the importance of the theoretical studies, lacking however in the systematic approach and ending up by providing data of corrosion inhibition of a crude extract, then performing computational studies on one only molecule of the extract itself [23,88]. This is clearly pointed up by the summary Table 1, reporting most of the papers cited in the present review and whether a computational/theoretical study has been included in the studies or not. The completeness of most of the papers lacks in this kind of approach, rarely ever focused on the mechanisms investigation and most frequently following a set of routine testing experiments.

## 5. Corrosion Inhibitors from Natural Sources

Organic chemicals operating as corrosion inhibitors were developed in the petroleum industry in the 1950s and were introduced to the concrete industry in the early 1990s. However, as already stated above, a major issue was immediately related to their toxicity. Usage of toxic materials as inhibitors was limited by many environmental care agencies because of their threat. As pointed out by NACE International report [102], methods of estimation the relationship between the structures of these inhibitors and their toxicity became necessary in the view of modifying the structure of the existing corrosion inhibitors to make these less toxic. The ideal and easiest approach to reduce the adverse effect of organic molecules is their substitution with less toxic alternatives. In this regard, there has been growing interest in the exploitation of natural extracts as inhibitors [77,88,103,104]. In addition to their environmentally friendly and ecologically acceptable features, many plant extracts are low-cost, readily available and renewable sources of materials of prospective industrial significance. These characteristics are justified by the abundant phytochemical constituents of the extracts, sharing many similarities with the molecular and electronic structures of conventional organic corrosion inhibitors, providing them the ability to adsorb onto metal surfaces. Extracts from their leaves, barks, seeds, fruits and roots comprise of mixtures of compounds containing heteroatoms often reported to function as effective inhibitors of metal corrosion in different aggressive environments. 

Already in 1981, Srivastava et al. published a paper highlighting that some natural compounds such as flour, gelatin, glue, gum, dextrin, industrial by-products, etc., have been reported as good corrosion inhibitors, pointing out how plant materials, such as leaves, fruits, flowers, roots and seeds, etc., had not yet been extensively tried for the same purpose [105]. The same authors then performed an experimental study on a large number of extracts from garlic, carrot, lignin, tobacco, and many others, in different corrosive environments. They made some preliminary hypotheses on the mechanisms. For example, they pointed out that the presence of S- or N-containing rings usually attach to the metal surface through the heteroatom. The electron density in the metal at the point of attachment may change, resulting in a retardation of the cathodic or anodic reactions. They evaluate the chemistry beyond the main constituent of each extract, setting up hypothesis of the adsorption mechanism. Their study clearly lacks in experimental and theoretical consistency and completeness, since there was neither any characterization of the single molecules of the extracts nor their respective inhibition effect. However, it was a useful starting point assessing the importance of crude extracts for this application. 

The mentioned lack of not providing a characterization of the extract is quite frequently encountered in the literature. This aspect comes up indeed from Table 1, reporting the main features of the extracts used in the literature as corrosion inhibitors. A column is dedicated to clarify if the authors performed a characterization of the extract in order to identify the main responsible of the inhibition or at least the most abundant molecules in the extract. This kind of characterization should be always performed, but in the reality is not. 

Aiming at citing and discussing some literature works on green inhibitors, it might be useful at this point to classify the most studied green inhibitors in the recent and past literature in order to rationalize their correspondent features depending on the chemistry beyond each class.

### 5.1. Amino Acids

The presence of amino acids in the panorama of natural corrosion inhibitors is clearly attributable mainly to the presence of nitrogen in all of their molecular structures. In addition, some of them—tyrosine, tryptophan and phenylalanine—contain aromatic rings that is one of the main factors decreasing the corrosion rate by blocking sites on the metal due to adsorption. Amino acids were widely reported as good safe corrosion inhibitors for many metals in various aggressive media. The inhibition process depends on both the nature of amino acid molecules and the state of the alloy surface. In neutral solutions, the amino acid molecules are present as Zwitterions. In chloride containing solutions, considered as pit initiators, the alloy surface is mostly covered with adsorbed layer of the Cl^−^. The amino acid molecule with its Zwitterion form will be adsorbed on the active sites, where the Cl^−^ are already present. The synergistic effect between the amino acid molecules and the chloride ions leads to adsorption of the amino acid molecules on the active sites and hence a decrease in the corrosion rate [92]. Of course, the pH of the solution affects the adsorption, due to the surface charge of the exposed metal surface. 

In the literature there are no works specifically focused on extracts containing amino acids in which a corrosion inhibition effect is attributed to specific amino acids. However, many papers documented the effectiveness of commercial amino acids addition to the corrosive environment. This should be regarded as a starting point to characterize these kinds of extracts.

Badawy et al. investigated the inhibition of a Ni-Cu alloy due to the presence in solution of some amino acids: glycine, alanine and leucine as aliphatic amino acids; cysteine as sulfur-containing amino acid; lysine and histidine as basic amino acids and glutamic acid as acid amino acid. The mechanism of the corrosion inhibition process was attributed to the adsorption of the amino acid on the active corrosion sites and the deposition of corrosion products with the formation of a 3D layer on the alloy surface. The inhibition efficiency hence depended on the properties of this layer [92]. The correlation between the presence of heteroatoms and the inhibition efficiency was studied by Kassou et al. [106]. Low carbon steel was studied in 200 ppm NaCl in the presence of Glycine, Cytosine, Thymine, Adenine and Guanine. The inhibition efficiency resulted following the order: Guanine > Adenine > Cytosine > Thymine > Glycine. This order can be explained by the heteroatoms number and type in these compounds. In fact, the Guanine has six heteroatoms and its inhibition is greater than the Adenine which has five heteroatoms, while Cytosine and Thymine have four heteroatoms only. Comparing Cytosine with Thymine, the inhibition efficiency is higher in the first compound, since Cytosine has three nitrogen atoms and one oxygen atom whereas the Thymine has two N atoms and two O atoms. This explains also that the nature of heteroatoms counts. However, this is a simplification of a more complex system, in which the efficiency of compounds depends upon electron density present around the heteroatoms, the number of adsorption active centers in the molecule and their charge density, molecular size, mode of adsorption, and formation of metallic complexes.

### 5.2. Alkaloids

Alkaloids constitute one of the widest class of natural products, being synthesized by many living organisms. Many research groups employed alkaloids extracts as green corrosion inhibitors due to the presence in their molecular structure of at least one nitrogen atom [65,70,83,91,96,97,107,108,109]. Due to the presence of lone pair electrons of heteroatoms and its interaction with the vacant d-orbital of the metal to be protected that are fundamental factors in the effective inhibition. 

The alkaloids extraction from the harvested matrix usually comprises a first extraction step of the dried matrix (pulverized or reduced to smaller coarse moieties) with solvents. If the matrix is particularly rich in oils and fats, these components should be eliminated before with a suitable non-polar solvent, such as hexane, which is unable to dissolve alkaloids. The dried products are then subjected to repeated acid-base treatment. In fact, alkaloids are first dissolved as salts by acidic solution exposure, and then alkaloidal salts are readily converted back to the corresponding alkaloid bases. A final organic solvent (immiscible with the aqueous phase) extraction, followed by solvent removal under reduced pressure, is the last step to obtain an alkaloids-enriched product. 

Many examples in the literature of natural products used as corrosion inhibitors followed indeed similar procedures, employing different matrices from plants. Raja et al. worked on the inhibition effect of *Neolamarckia cadamba* crude extract (bark, leaves) and pure alkaloid (3β-isodihydrocadambine) for mild steel corrosion in 1 M HCl medium [65]. Leaves and bark were first defatted with hexane and, after drying, moistened in 25% NH_4_OH, soaked and macerated with CH_2_Cl_2_ and finally the crude extracts were subjected to acid-base treatment to obtain alkaloid matrix from leaves and from bark. Further purification and elucidation of the main compound, 3β-isodihydrocadambine, was performed. Three samples were then tested as corrosion inhibitors: leaves extract, bark extract and the purified 3β-isodihydrocadambine. FTIR analysis suggested that the responsible of adsorption, and hence of the inhibition, was the aromatic indole ring and carbonyl group of 3β-isodihydrocadambine, involved in coordination with the metallic surface. Molecular modelling studies supported the FTIR findings. A valuable attempt to discriminate the role of different components of the extract was made. However, the discussion, once again, does not conclude anything about any synergistic role of the compounds, which were not exhaustively identified. In the same period, in another work by Faustin and co-workers, the corrosion inhibition by alkaloids extract from *Geissospermum laeve* on C38 steel in 1 M HCl was documented [83]. *Geissospermum laeve* bark was collected, dried and extracted by acid-base procedures. In the optic of identifying the main responsible of inhibition, the major alkaloid of the extract (constituting the 3.5% by weight), geissospermine, was isolated and characterized. Electrochemical tests were hence run using, as a first sample, the alkaloids extract and, as a second one, the geissospermine molecules at the same concentration as what contained in the extract. Results indeed confirm that the geissospermine molecule was the constituent responsible for the corrosion inhibition of the entire extract. The authors then discussed some hypotheses about the mechanism of corrosion inhibition depending on the protonation of the molecule and hence its charge distribution on the molecule. Fdil and co-authors published two papers, in 2015 [97] and 2018 [96], about alkaloids extract of *Retama monosperma* (L.) *Boiss.* seeds and stems, used as novel eco-friendly inhibitor for carbon steel corrosion in 1 M HCl solution. In the first paper the main alkaloids constituting the extract were identified in cytisine with a percentage of 78%, together with minor amounts of dehydro-cytisine (9.37%) and N-methylcytisine (13%). In the stems extract, discussed in the second work, the main alkaloids were sparteine (29%), ammodendrine (24%), anagyrine (12%) and dehydrosparteine (9%). A fine characterization of single-compound inhibition was not carried out and this clearly renders difficult to make any hypothesis about the details of the mechanisms. The characterization of the alkaloids of the extract is less studied in many works. Djemoui investigated the inhibition effect of the alkaloids extract of *Peganum harmala* L. (Syrian rue), on the corrosion of 6063 aluminum alloy in 1 M HCl solution. The usual composition of the extract (harmine and harmaline) is mentioned in the paper introduction but neither relative percentage nor specific characterization of the extract was performed [70]. Another work, focusing on alkaloids extract from *Palicourea guianensis* plant as corrosion inhibitor for C38 Steel in 1 M HCl, after extraction of the alkaloids, described an attempt to separate the constituents but, realizing the great number of compounds, ended up by not characterizing any of them and using the total extract [91]. 

### 5.3. Phenols and Polyphenols

Phenols and polyphenols are regarded as other classes of important natural compounds found ubiquitous in plants. Their corrosion inhibitive action is mainly attributed to the presence of aromatic hydroxyl groups. This is again explained by the ability of heteroatoms to coordinate bonds with vacant d orbitals of metal through electrons donation. Also, the interactions with rings containing conjugated bonds, π electrons, enable adsorption of molecules on metal surface. El-Etre published a paper on the corrosion inhibition of carbon steel by olive leaves extracts. Dry olive leaves were boiled in water for 3 h and, after filtrate evaporation, the solid residue was used to prepare the desired solutions of inhibitors. The inhibitive phenomenon toward the acid corrosion of steel was attributed to the adsorption through the lone pairs of electrons of the oxygen atoms forming a covering film, acting as a barrier between the steel surface and the aggressive solution. In addition, the arrangement of oxygen atoms surrounding the aromatic rings of the phenols, may lead to the conclusion that phenolic compounds are forced to be adsorbed horizontally onto the steel surface. The aqueous extract of olive (*Olea europaea* L.) leaves contains polyphenolic compounds with antioxidant potential. The major constituents were identified in: oleuropein and hydroxytyrosol. The other polyphenols present include tyrosol, oleuropein aglycone, and gallic acid. Since oleuropein is readily hydrolyzed to hydroxytyrosol and elenolic acid, hydroxytyrosol might be the main responsible of the inhibition process. However, there was no experimental proof confirming these hypothesis about the inhibition action mechanism and the activity of single compounds were not verified in the work [98]. In a more recent paper, an evaluation of polyphenol composition and anticorrosion properties of mild steel was carried out starting from the *Cryptostegia grandiflora* plant methanol extract. The extract showed the highest concentration of myricetin, quercetin, and rutin but the single inhibition activities are missing [99]. A different approach was adopted by Abdallah and co-workers, selecting four different commercial phenols, and evaluating their single inhibition activities towards the corrosion of steel. The molecules were characterized by the presence of amine, hydroxyl, aldehyde, or carboxyl groups in ortho-position with respect to the phenol. The order of inhibition efficiency was the following: -NH_2_ > -OH > -CHO > -COOH. From the sequence it was derived that, compounds containing electron donating groups are more efficient as inhibitors than compounds containing electron withdrawing groups. The electron donating groups enhance adsorption and add electron density to the π system making it more nucleophilic and consequently, increase the inhibition efficiency. Theoretical calculations about HOMO-LUMO orbitals were also provided [110]. In another work by Wei Tan et al., nine different solvents were employed in the extraction of phenols from *Rhizophora apiculata*. The total phenolic content extracted by the solvents was in the descending order of 70% acetone > ethanol-water 1:1 > ethanol-water 1:2 > water at 90 °C > ethanol > water at 50 °C > water > acetone > isopropyl alcohol, suggesting that 70% acetone gave the highest yield of phenolic content. A relation between phenolic profiles and inhibitive properties of the extract was identified. The inhibitive properties of *Rhizophora apiculata* was attributed to the presence of condensed tannin in the extracts [100]. 

An interesting experimental work by another group, pointed out that a relationship exists between the scavenging effect of polyphenols and their anticorrosive properties. Their results showed that the greater the mass of molecules and the higher the number of ortho-OH groups, the better the properties shown by the polyphenol as a scavenger of DPPH and as a rust converter. Molecular masses were plotted versus the protection efficiencies. Protection efficiency of the rusts indeed increased with increases in the molecular mass of the polyphenol and the content of OH groups in it [111]. The scavenging effect of polyphenols may hence be used to evaluate and predict the applicability of various polyphenols to anticorrosive purposes.

Some works in the literature prepared extracts from different part of the *Punica granatum* plant, such as seeds [107] and peel [112]. Among the main phytochemical constituents, gallic acid, tannic acid and ellagic acid were identified as the predominant ones. Seeds were extracted with refluxing ethanol, peel with methanol. It was stated that the great amount of constituents of the extracts made it rather difficult to assign the observed corrosion inhibiting effect to any particular molecule. An attempt to build a theoretical model was provided, together with the prediction of mechanisms of adsorption of the main molecules. The lack, however, is still the absence of any simplified experimental approach that could be able to discriminate the single compounds contribution to the overall activity.

### 5.4. Fatty Acids

The importance of this class for the inhibition of corrosion is due to molecular features of these compounds. Plausibly, the unsaturated bonds and the presence of active functional group in fatty acids (carboxylate anion) increase the surface chemistry toward interaction with metal-vacant orbitals and play a key role to enhance the adsorption characteristics. The carboxylate anion of fatty acid molecules may interact with the metal causing the formation of metal−inhibitor complex and therefore a decrease of the dissolution rate of metallic surface in the corrosive medium, owing to a retarded hydrogen evolution reaction, for example. Moreover, the presence of conjugated and non-conjugated double bonds in fatty acids could be able to share the localized π electron with vacant d orbital of metals. Additionally, binding of high-molecular-weight fatty acids over the metal decreases the available surface due to steric hindrance, addressing the declination of corrosion. A very recent work by Khanra and co-workers, was focused on the extraction of unsaturated fatty acids from a microalga, *Scenedesmus* sp., and its application toward mild steel corrosion inhibition in HCl solution [101]. As microalgae are abundant and a sustainable source of oil accumulation, microalgal oil has been introduced to elucidate the anticorrosion proficiency of mild steel in acidic medium. After centrifugation, cell pellets were dried and then the fatty acids were extracted with a mixture 2:1 of chloroform and methanol. The mixture was ultrasonicated and the supernatant was collected after centrifugation. Distilled water was added for phase separation, and the lower organic phase was recovered and dried in a rotary evaporator. The organic phase, containing fatty acids, was utilized as inhibitor. By GC-MS, the major fatty acids were identified in hexadecanoic acid, 9,12-octadecadieonic acid and 9,12,15-octadecatrienoic acid. Among the fatty acid constituents, total saturated fatty acid and unsaturated fatty acid were found to be nearly 35 and 48%, respectively. By quantum chemical calculation studies, the primary inhibition efficiency was assigned to 9,12,15-octadecatrienoic acid and corrosion inhibition potential of the tested fatty acids was found to follow the trend C18:3 > C18:2 > C16:0. The contribution of unsaturated molecules like 9,12,15-octadecatrienoic acid and 9,12-octadecadienoic acid toward the corrosion inhibition should be higher compared to that of saturated molecule, for the reasons above explained. These last speculations were derived from computational/mechanistic studies. An experimental support to these hypotheses, by addition of single fatty acids compounds to the corrosive media, could validate the work by discriminating the contributions from single molecules. 

## 6. Corrosion Inhibitors from Biomass Waste

The importance of working on bio-wastes in a circular economy context is crucial in a background like the one of the present review. Only a few works, among the great number of previously mentioned papers, dealt with extracts from natural residues and bio-wastes, which are generally discarded in the environment [11,12,13,16,58,89]. About these last cited papers: Fiori-Bimbi et al. [16] and Fares et al. [58] started from pectin derived from citrus peel; Hussein et al. extracted chitosan from seafood waste [89]; Ismail et al. used fresh leaves of plants grown for fruits (banana, sugarcane and watermelon) [11]; Odewunmi et al. started from rind, seed and peel of watermelons [12]; Grassino et. al. employed tomato derived by-products as sources of pectin [13]. It is anyway interesting how, in these few mentioned works, authors selected waste-based matrices but most of them neglected to highlight this aspect in their introduction as if they were not aware of the importance of the topic. Only Grassino and co-authors pointed out the importance not only to start from bio-based sources, but also to select by-products and wastes and utilizing them as a cheap source of different bioactive compounds [13]. In their paper, by-products composed of tomato peels, seeds and small amount of pulp, usually disposed as a solid waste or used as animal feed, have been employed as pectin sources for the corrosion inhibition of tin. Considering two works in which pectin from citrus peel was employed [16,58], authors were indeed employing food wastes, without a complete awareness of the importance of the matrix they were using. In a recent review on citrus peel waste utilization for other applications, the authors pointed out how wastes are capable of offering significant compounds at low-cost and easy availability [113]. Utilization of these bioactive residues provides an efficient, inexpensive, and environment-friendly platform for the production of useful compounds.

The great number of works dealing with natural compounds as corrosion inhibitors, on the other side, did not pay any kind of attention to this aspect. Evidently, the circular economy context and principles have not affected this field yet. In Table 1, that summarizes all the features of natural products employed as green corrosion inhibitors cited in the present review, one column is dedicated to pointing out whether the starting source of corrosion inhibitors is derived from bio-wastes. Most of the cited paper employed high-value sources. 

The future research in the field might be addressed in the directions of a broader circular economy background with a careful evaluation of the starting source, coming to terms with the value of the source itself.

## Figures and Tables

**Table 1:** Summary of the main features of the cited green extracts employed as corrosion inhibitors.

Plant or Source	Is the Source Derived from Bio-Wastes?	Major Constituent Responsible for Inhibition	Solvent and Extraction Method	Metal to Protect	Corrosive Environment	Max Corrosion Inhibition Efficiency/%	Inhibitor Concetration	Theoretical Calculations	Reference
Propolis	no	n.d	Boiling water	Carbon steel	10 ppm NaCl and 35 ppm Al_2_ (SO_4_)_3_	92.7 at 30 °C	0.6 g L^−1^	n.r.	[28]
11% *w*/*v* ethanolic propolis extract	no	3,5-diprenyl-4-hydroxycinnamic acid	Commercial	Mild steel	3.5% *w*/*v* NaCl	n.r.	13 ± 3 g m^−2^	n.r.	[29]
Citrus peel	yes	Pectin	HCl hydrolysis-precipitation with ethanol	Mild steel	1 M HCl	94.2 at 45 °C	2 g L^−1^	n.r.	[16]
Citrus peel	yes	Pectin	Commercial	Aluminum	0.5 to 2 M HCl	91 at 10 °C	8 g L^−1^	n.r.	[58]
Pectin from apples	no	Pectin	Commercial	X60 steel	0.5 M HCl	78.7 at 25 °C	1 g L^−1^	yes	[26]
Pectin from apples	no	Pectin and CeO_2_	Commercial	X60 steel	0.5 M HCl	75.4 at 25 °C	0.5 g L^−1^ pectin + 5 mM CeO_2_	n.r.	[27]
Cladodes of *Opuntia Ficus Indica*	no	Pectin	Microwave	Steel	1 M HCl	94 at 35 °C	1 g L^−1^	n.r.	[94]
Leaves of henna	no	n.d.	Boiling water	C-steel	0.1 M HCl	95.78 at 30 °C	0.8 g L^−1^	n.r.	[49]
					3.5% NaCl	91.01 at 30 °C			
					0.1 M NaOH	69.56 at 30 °C			
				Nickel	0.1 M HCl	88.77 at 30 °C			
					3.5% NaCl	82.88 at 30 °C			
					0.1 M NaOH	73.91 at 30 °C			
				Zinc	0.1 M HCl	76.19 at 30 °C			
					3.5% NaCl	93.44 at 30 °C			
					0.1M NaOH	76.92 at 30 °C			
Four brands of leaves of henna	no	n.d.	Shaking in the corrosive medium	Aluminium and steel	0.1 M HCl, 0.1 M NaOH and 0.1 M NaCl	n.r.	n.r.	n.r.	[81]
Leaves of henna	yes	Lawsone, Gallic acid, α-d-Glucose and Tannic acid	Boiling water	Mild steel	1 M HCl	90.34 at 25 °C	1.2 g L^−1^	yes	[77]
*Salvia hispanica* seeds	no	n.d.	Soaking in methanol	Bronze	Simulated acid rain solution	95	0.4 g L^−1^	n.r.	[60]
Fresh leaves of *Justicia gendarussa*	no	n.d.	Methanol	Mild steel	1 M HCl	91.6 at 25 °C	0.15 g L^−1^	Attempts	[68]
Grains of *Peganum harmala*	no	n.d.	Methanol, then acidic/alkaline extraction	6063 Aluminium Alloy	1 M HCl	91.78 at 25 °C	0.025 g L^−1^	n.r.	[70]
*Schinopsis lorentzii* tree powder	yes	Flavan-3-ol	ASTM 1110-96 and TAPPI T204 OM-88 standard	Low carbon steel	1 M HCl	66	0.2 g L^−1^	Attempts	[71]
Ascorbic acid and folic acid	no	Ascorbic acid and folic acid	Commercial	Mild steel	0.3 and 0.03% NaCI	n.d.	0.2 g L^−1^ for ascorbic acid and 0.5 g L^−1^ for folic acid	yes	[73]
Ascorbic acid	no	Ascorbic acid	Commercial	Mild steel	H_2_SO_4_ pH 4	71.5 at 20 °C	10^−3^ mol L^−1^	n.r.	[66]
Seeds of *Piper guineense*	no	Piperine, Safrole, and Dihydrocubebin	Refluxing in 1 M HCl	Mild steel	1 M HCl	88.4	0.9 g L^−1^	yes	[76]
			Refluxing in 0.5 M H_2_SO_4_		0.5 M H_2_SO_4_	97.7	0.9 g L^−1^		
Streptomycin	no	Streptomycin	Commercial	Mild steel	1 M HCl	88.5 at 35 °C	0.5 g L^−1^	n.r.	[78]
*Aspidosperma album* bark	no	n.d.	Acidic/alkaline extraction	C38 steel	1 M HCl	90 at 25 °C	0.1 g L^−1^	n.r.	[82]
*Geissospermum* laeve	no	Geissospermine	Acidic/alkaline extraction	C38 steel	1 M HCl	90 at 25 °C	0.1 g L^−1^	yes	[83]
*Retama monosperma* (L.) *Boiss.* stems	no	Sparteine, ammodendrine, anagyrin and dehydrosparteine	Acidic/alkaline extraction	Carbon steel	1 M HCl	83 at 30 °C	0.4 g L^−1^	n.r.	[96]
*Retama monosperma* (L.) *Boiss.* seeds	no	Cytisine	Acidic/alkaline extraction	Carbon steel	1 M HCl	94.42 at 30 °C	0.4 g L^−1^	n.r.	[97]
Bark and leaves of *Neolamarckia cadamba*	no	3β-isodihydrocadambine	Acidic/alkaline extraction	Mild steel	1 M HCl	91 at 30 °C	0.005 g L^−1^ of bark extract	yes	[65]
Leaves of *Palicourea guianensis*	no	n.d.	Acidic/alkaline extraction	C38 steel	1 M HCl	89 at 25 °C	0.1 g L^−1^	n.r.	[91]
Dry olive leaves	yes	Hydroxytyrosol (hypothesis)	Boiling water	Carbon steel	2 M HCl	93 at 25 °C	0. 9 g L^−1^	n.r.	[98]
Leaves of *Cryptostegia grandiflora*	no	Myricetin, quercetin, and rutin	Soaking in methanol	Mild steel	1 M H_2_SO_4_	83.54 at 30 °C	0.5 g L^−1^	n.r.	[99]
Bark of *Rhizophora apiculata*	no	n.d.	Maceration in 9 different solvents	Mild steel	1 M HCl	57.9	0.1 g L^−1^	n.r.	[100]
*Scenedemus* alge	no	9,12,15-octadecatrienoic acid (C18:3); 9,12-octadecadienoic acid (C18:2), and hexadecanoic acid (C16:0)	Chloroform and methanol	Mild steel	1 M HCl	95.1	0.035 g L^−1^	yes	[101]
Tomato peel waste	yes	Pectin	Extraction with oxalates	Tin	2% NaCl, 1% acetic acid and 0.5% citric acid solution	72.98 at 25 °C	4 g L^−1^	yes	[13]
Solid waste from fresh leaves of Banana, sugarcane and wate melon hardcore	yes	n.d.	Ethanol	Mild steel	1 M HCl	69.60, 68.41 and 58.15 at 25 °C for Banana, sugarcane and watermelon hardcore	10% *v*/*v*	n.r.	[11]
Shrimps shell waste	yes	Chitosan	NaOH	Carbon steel	1 M HCl	88.50 at 25 °C	10^−5^ M	n.r.	[89]
Watermelon rind, seeds and peel	yes	n.d.	Boiling in 1 M HCl	Mild steel	1 M HCl	Max of 86.08 for seeds extract	2 g L^−1^	Attempts	[12]

n.d.: not determined; n.r.: not reported.
